# Supplementary material for: Artificial Intelligence-Enhanced Molecular Profiling of JAK-STAT Pathway Alterations in FOLFOX-Treated Early-Onset Colorectal Cancer
Source: Int J Mol Sci. 2026 Jan 2;27(1):479. doi: 10.3390/ijms27010479 (PMC12786996; doi:10.3390/ijms27010479)
Supplement: Supplementary file 1 [file ijms-27-00479-s001.zip › ijms-4041779-supplementary.pdf]

Supplementary Materials:

Table S1 - Comparison of Early-Onset Hispanic/Latino (H/L) Patients Treated with FOLFOX versus Not Treated with FOLFOX

JAK/STAT Pathway			
**Gene**	**Early-Onset Hispanic/Latino Treated with FOLFOX n (%)**	**Early-Onset Hispanic/Latino Not Treated with FOLFOX n (%)**	**p-value**
**JAK1 Mutation**			
Present	1 (1.4%)	4 (7.7%)	0.1594
Absent	72 (98.6%)	48 (92.3%)	
**JAK2 Mutation**			
Present	0 (0.0%)	0 (0.0%)	1
Absent	73 (100.0%)	52 (100.0%)	
**JAK3 Mutation**			
Present	1 (1.4%)	3 (5.8%)	0.3067
Absent	72 (98.6%)	49 (94.2%)	
**SOCS1 Mutation**			
Present	1 (1.4%)	0 (0.0%)	1
Absent	72 (98.6%)	52 (100.0%)	
**STAT3 Mutation**			
Present	0 (0.0%)	2 (3.8%)	0.1711
Absent	73 (100.0%)	50 (96.2%)	
**STAT5A Mutation**			
Present	0 (0.0%)	2 (3.8%)	0.1711
Absent	73 (100.0%)	50 (96.2%)	
**STAT5B Mutation**			
Present	0 (0.0%)	5 (9.6%)	**0.01108**
Absent	73 (100.0%)	47 (90.4%)	

Table S2 - Comparison of Late-Onset Hispanic/Latino (H/L) Patients Treated with FOLFOX versus Not Treated with FOLFOX

JAK/STAT Pathway			
Gene	Late-Onset Hispanic/Latino Treated with FOLFOX n (%)	Late-Onset Hispanic/Latino Not Treated with FOLFOX n (%)	p-value
**JAK1 Mutation**			
Present	2 (2.2%)	2 (2.2%)	1
Absent	89 (97.8%)	89 (97.8%)	
**JAK2 Mutation**			
Present	2 (2.2%)	2 (2.2%)	1
Absent	89 (97.8%)	89 (97.8%)	
**JAK3 Mutation**			
Present	4 (4.4%)	4 (4.4%)	1
Absent	87 (95.6%)	87 (95.6%)	
**SOCS1 Mutation**			
Present	0 (0.0%)	0 (0.0%)	1
Absent	91 (100.0%)	91 (100.0%)	
**STAT3 Mutation**			
Present	1 (1.1%)	1 (1.1%)	1
Absent	90 (98.9%)	90 (98.9%)	
**STAT5A Mutation**			
Present	2 (2.2%)	2 (2.2%)	1
Absent	89 (97.8%)	89 (97.8%)	
**STAT5B Mutation**			
Present	1 (1.1%)	1 (1.1%)	1
Absent	90 (98.9%)	90 (98.9%)	

Table S3 - Comparison of Early-Onset Non-Hispanic White (NHW) Patients Treated with FOLFOX versus Not Treated with FOLFOX

JAK/STAT Pathway			
Gene	Early-Onset NHW Treated with FOLFOX n (%)	Early-Onset NHW Not Treated with FOLFOX n (%)	p-value
**JAK1 Mutation**			
Present	10 (2.7%)	10 (3.3%)	0.7917
Absent	365 (97.3%)	292 (96.7%)	
**JAK2 Mutation**			
Present	5 (1.3%)	9 (3.0%)	0.2206
Absent	370 (98.7%)	293 (97.0%)	
**JAK3 Mutation**			
Present	4 (1.1%)	14 (4.6%)	**0.006502**
Absent	371 (98.9%)	288 (95.4%)	
**SOCS1 Mutation**			
Present	1 (0.3%)	1 (0.3%)	1
Absent	374 (99.7%)	301 (99.7%)	
**STAT3 Mutation**			
Present	7 (1.9%)	3 (1.0%)	0.5245
Absent	368 (98.1%)	299 (99.0%)	
**STAT5A Mutation**			
Present	3 (0.8%)	5 (1.7%)	0.4772
Absent	372 (99.2%)	297 (98.3%)	
**STAT5B Mutation**			
Present	5 (1.3%)	7 (2.3%)	0.5015
Absent	370 (98.7%)	295 (97.7%)	

Table S4 - Comparison of Late-Onset Non-Hispanic White (NHW) Patients Treated with FOLFOX versus Not Treated with FOLFOX

JAK/STAT Pathway			
Gene	Late-Onset NHW Treated with FOLFOX n (%)	Late-Onset NHW Not Treated with FOLFOX n (%)	p-value
**JAK1 Mutation**			
Present	10 (1.8%)	39 (6.0%)	0.1151
Absent	292 (98.2%)	614 (94.0%)	
**JAK2 Mutation**			
Present	9 (1.2%)	17 (2.6%)	0.9054
Absent	293 (98.8%)	636 (97.4%)	
**JAK3 Mutation**			
Present	14 (2.6%)	28 (4.3%)	0.9409
Absent	288 (97.4%)	625 (95.7%)	
**SOCS1 Mutation**			
Present	1 (0.4%)	2 (0.3%)	1
Absent	301 (99.6%)	651 (99.7%)	
**STAT3 Mutation**			
Present	3 (1.5%)	11 (1.7%)	0.5666
Absent	299 (98.5%)	642 (98.3%)	
**STAT5A Mutation**			
Present	5 (0.7%)	9 (1.4%)	0.9664
Absent	297 (99.3%)	644 (98.6%)	
**STAT5B Mutation**			
Present	7 (0.9%)	21 (3.2%)	0.5764
Absent	295 (99.1%)	632 (96.8%)	

Table S5 - Comparison of Early-Onset versus Late-Onset Hispanic/Latino (H/L) Patients Treated with FOLFOX

JAK/STAT Pathway			
Gene	Early-Onset Hispanic/Latino Treated with FOLFOX n (%)	Late-Onset Hispanic/Latino Treated with FOLFOX n (%)	p-value
**JAK1 Mutation**			
Present	1 (1.4%)	2 (2.2%)	1
Absent	72 (98.6%)	89 (97.8%)	
**JAK2 Mutation**			
Present	0 (0.0%)	2 (2.2%)	0.503
Absent	73 (100.0%)	89 (97.8%)	
**JAK3 Mutation**			
Present	1 (1.4%)	4 (4.4%)	0.3826
Absent	72 (98.6%)	87 (95.6%)	
**SOCS1 Mutation**			
Present	1 (1.4%)	0 (0.0%)	0.4451
Absent	72 (98.6%)	91 (100.0%)	
**STAT3 Mutation**			
Present	0 (0.0%)	1 (1.1%)	1
Absent	73 (100.0%)	90 (98.9%)	
**STAT5A Mutation**			
Present	0 (0.0%)	2 (2.2%)	0.503
Absent	73 (100.0%)	89 (97.8%)	
**STAT5B Mutation**			
Present	0 (0.0%)	1 (1.1%)	1
Absent	73 (100.0%)	90 (98.9%)	

Table S6 - Comparison of Early-Onset versus Late-Onset Hispanic/Latino (H/L) Patients Not Treated with FOLFOX

JAK/STAT Pathway			
**Gene**	**Early-Onset Hispanic/Latino Not Treated with FOLFOX n (%)**	**Late-Onset Hispanic/Latino Not Treated with FOLFOX n (%)**	**p-value**
**JAK1 Mutation**			
Present	4 (7.7%)	2 (2.2%)	0.1903
Absent	48 (92.3%)	89 (97.8%)	
**JAK2 Mutation**			
Present	0 (0.0%)	2 (2.2%)	0.5339
Absent	52 (100.0%)	89 (97.8%)	
**JAK3 Mutation**			
Present	3 (5.8%)	4 (4.4%)	0.705
Absent	49 (94.2%)	87 (95.6%)	
**SOCS1 Mutation**			
Present	0 (0.0%)	0 (0.0%)	1
Absent	52 (100.0%)	91 (100.0%)	
**STAT3 Mutation**			
Present	2 (3.8%)	1 (1.1%)	0.2992
Absent	50 (96.2%)	90 (98.9%)	
**STAT5A Mutation**			
Present	2 (3.8%)	2 (2.2%)	0.6218
Absent	50 (96.2%)	89 (97.8%)	
**STAT5B Mutation**			
Present	5 (9.6%)	1 (1.1%)	**0.02405**
Absent	47 (90.4%)	90 (98.9%)	

Table S7 - Comparison of Early-Onset versus Late-Onset Non-Hispanic White (NHW) Patients Treated with FOLFOX

JAK/STAT Pathway			
**Gene**	**Early-Onset NHW Treated with FOLFOX n (%)**	**Late-Onset NHW Treated with FOLFOX n (%)**	**p-value**
**JAK1 Mutation**			
Present	10 (2.7%)	10 (1.8%)	0.7917
Absent	365 (97.3%)	292 (98.2%)	
**JAK2 Mutation**			
Present	5 (1.3%)	9 (1.2%)	0.2206
Absent	370 (98.7%)	293 (98.8%)	
**JAK3 Mutation**			
Present	4 (1.1%)	14 (2.6%)	**0.006502**
Absent	371 (98.9%)	288 (97.4%)	
**SOCS1 Mutation**			
Present	1 (0.3%)	1 (0.4%)	1
Absent	374 (99.7%)	301 (99.6%)	
**STAT3 Mutation**			
Present	7 (1.9%)	3 (1.5%)	0.5245
Absent	368 (98.1%)	299 (98.5%)	
**STAT5A Mutation**			
Present	3 (0.8%)	5 (0.7%)	0.4772
Absent	372 (99.2%)	297 (99.3%)	
**STAT5B Mutation**			
Present	5 (1.3%)	7 (0.9%)	0.5015
Absent	370 (98.7%)	295 (99.1%)	

Table S8 - Comparison of Early-Onset Hispanic/Latino (H/L) versus Early-Onset Non-Hispanic White (NHW) Patients Treated with FOLFOX

JAK/STAT Pathway			
Gene	Early-Onset Hispanic/Latino Treated with FOLFOX n (%)	Early-Onset NHW Treated with FOLFOX n (%)	p-value
**JAK1 Mutation**			
Present	1 (1.4%)	10 (2.7%)	1
Absent	72 (98.6%)	365 (97.3%)	
**JAK2 Mutation**			
Present	0 (0.0%)	5 (1.3%)	1
Absent	73 (100.0%)	370 (98.7%)	
**JAK3 Mutation**			
Present	1 (1.4%)	4 (1.1%)	0.5909
Absent	72 (98.6%)	371 (98.9%)	
**SOCS1 Mutation**			
Present	1 (1.4%)	1 (0.3%)	0.2996
Absent	72 (98.6%)	374 (99.7%)	
**STAT3 Mutation**			
Present	0 (0.0%)	7 (1.9%)	0.6049
Absent	73 (100.0%)	368 (98.1%)	
**STAT5A Mutation**			
Present	0 (0.0%)	3 (0.8%)	1
Absent	73 (100.0%)	372 (99.2%)	
**STAT5B Mutation**			
Present	0 (0.0%)	5 (1.3%)	1
Absent	73 (100.0%)	370 (98.7%)	

Table S9 - Comparison of Early-Onset Hispanic/Latino (H/L) versus Early-Onset Non-Hispanic White (NHW) Patients Not Treated with FOLFOX

JAK/STAT Pathway			
Gene	Early-Onset Hispanic/Latino Not Treated with FOLFOX n (%)	Early-Onset NHW Not Treated with FOLFOX n (%)	p-value
**JAK1 Mutation**			
Present	4 (7.7%)	10 (3.3%)	0.1342
Absent	48 (92.3%)	292 (96.7%)	
**JAK2 Mutation**			
Present	0 (0.0%)	9 (3.0%)	0.3669
Absent	52 (100.0%)	293 (97.0%)	
**JAK3 Mutation**			
Present	3 (5.8%)	14 (4.6%)	0.7245
Absent	49 (94.2%)	288 (95.4%)	
**SOCS1 Mutation**			
Present	0 (0.0%)	1 (0.3%)	1
Absent	52 (100.0%)	301 (99.7%)	
**STAT3 Mutation**			
Present	2 (3.8%)	3 (1.0%)	0.158
Absent	50 (96.2%)	299 (99.0%)	
**STAT5A Mutation**			
Present	2 (3.8%)	5 (1.7%)	0.2743
Absent	50 (96.2%)	297 (98.3%)	
**STAT5B Mutation**			
Present	5 (9.6%)	7 (2.3%)	**0.01994**
Absent	47 (90.4%)	295 (97.7%)	

Table S10 - Comparison of Late-Onset Hispanic/Latino (H/L) versus Late-Onset Non-Hispanic White (NHW) Patients Treated with FOLFOX

JAK/STAT Pathway			
Gene	Late-Onset Hispanic/Latino Treated with FOLFOX n (%)	Late-Onset NHW Treated with FOLFOX n (%)	p-value
**JAK1 Mutation**			
Present	2 (2.2%)	10 (1.8%)	0.7407
Absent	89 (97.8%)	292 (98.2%)	
**JAK2 Mutation**			
Present	2 (2.2%)	9 (1.2%)	1
Absent	89 (97.8%)	293 (98.8%)	
**JAK3 Mutation**			
Present	4 (4.4%)	14 (2.6%)	1
Absent	87 (95.6%)	288 (97.4%)	
**SOCS1 Mutation**			
Present	0 (0.0%)	1 (0.4%)	1
Absent	91 (100.0%)	301 (99.6%)	
**STAT3 Mutation**			
Present	1 (1.1%)	3 (1.5%)	1
Absent	90 (98.9%)	299 (98.5%)	
**STAT5A Mutation**			
Present	2 (2.2%)	5 (0.7%)	0.665
Absent	89 (97.8%)	297 (99.3%)	
**STAT5B Mutation**			
Present	1 (1.1%)	7 (0.9%)	0.6875
Absent	90 (98.9%)	295 (99.1%)	

Table S11 - Comparison of Late-Onset Hispanic/Latino (H/L) versus Late-Onset Non-Hispanic White (NHW) Patients Not Treated with FOLFOX

JAK/STAT Pathway			
Gene	Late-Onset Hispanic/Latino Not Treated with FOLFOX n (%)	Late-Onset NHW Not Treated with FOLFOX n (%)	p-value
**JAK1 Mutation**			
Present	2 (2.2%)	39 (6.0%)	0.2158
Absent	89 (97.8%)	614 (94.0%)	
**JAK2 Mutation**			
Present	2 (2.2%)	17 (2.6%)	1
Absent	89 (97.8%)	636 (97.4%)	
**JAK3 Mutation**			
Present	4 (4.4%)	28 (4.3%)	1
Absent	87 (95.6%)	625 (95.7%)	
**SOCS1 Mutation**			
Present	0 (0.0%)	2 (0.3%)	1
Absent	91 (100.0%)	651 (99.7%)	
**STAT3 Mutation**			
Present	1 (1.1%)	11 (1.7%)	1
Absent	90 (98.9%)	642 (98.3%)	
**STAT5A Mutation**			
Present	2 (2.2%)	9 (1.4%)	0.6332
Absent	89 (97.8%)	644 (98.6%)	
**STAT5B Mutation**			
Present	1 (1.1%)	21 (3.2%)	0.5035
Absent	90 (98.9%)	632 (96.8%)	

Table S12 - Patterns of JAK-STAT Pathway Mutation Types Across Ancestry, Age-of-Onset Groups, and FOLFOX Treatment in Colorectal Cancer. This table summarizes the distribution of mutation types observed in JAK-STAT pathway genes, categorized by ancestry [Hispanic/Latino (H/L) and Non-Hispanic White (NHW)], diagnostic age group [early-onset (EO) vs. late-onset (LO)], and FOLFOX treatment exposure (treated vs. untreated). Mutation classes include frameshift insertions and deletions, in-frame indels, missense and nonsense mutations, nonstop variants, splice site and splice-region alterations, and changes affecting translation initiation. The percentages shown represent the relative contribution of each mutation class to the total mutations identified for each

gene within the specified subgroup. This table provides a comparative view of variation in mutation spectra across demographic and clinical strata, enabling assessment of how ancestry, age, and treatment status relate to differences in the types of JAK-STAT alterations detected.

	Hispanic/Latino Samples				Non-Hispanic White Samples			
	Early-Onset		Late-Onset		Early-Onset		Late-Onset	
	Treated with FOLFOX	Not Treated with FOLFOX	Treated with FOLFOX	Not Treated with FOLFOX	Treated with FOLFOX	Not Treated with FOLFOX	Treated with FOLFOX	Not Treated with FOLFOX
**JAK1**								
Frame Shift Deletion	100.0%	7.1%	33.3%	100.0%	50.0%	0.0%	27.3%	46.0%
Frame Shift Insertion	0.0%	7.1%	33.3%	0.0%	7.1%	9.1%	4.5%	4.0%
In Frame Deletion	0.0%	0.0%	0.0%	0.0%	0.0%	0.0%	4.5%	2.0%
Missense Mutation	0.0%	28.6%	33.3%	0.0%	42.9%	81.8%	54.5%	44.0%
Nonsense Mutation	0.0%	14.3%	0.0%	0.0%	0.0%	0.0%	0.0%	2.0%
Splice Site	0.0%	42.9%	0.0%	0.0%	0.0%	9.1%	9.1%	2.0%
**JAK2**								
Frame Shift Deletion	0.0%	0.0%	0.0%	0.0%	0.0%	8.3%	15.4%	10.5%
Frame Shift Insertion	0.0%	0.0%	0.0%	0.0%	20.0%	0.0%	0.0%	0.0%
Missense Mutation	0.0%	0.0%	50.0%	0.0%	80.0%	75.0%	84.6%	73.7%
Nonsense Mutation	0.0%	0.0%	50.0%	0.0%	0.0%	16.7%	0.0%	15.8%
**JAK3**								
Frame Shift Deletion	0.0%	0.0%	0.0%	0.0%	25.0%	12.5%	12.0%	10.0%
Frame Shift Insertion	0.0%	0.0%	0.0%	0.0%	0.0%	0.0%	4.0%	6.7%
In Frame Deletion	0.0%	0.0%	25.0%	0.0%	0.0%	0.0%	0.0%	0.0%
Missense Mutation	100.0%	66.7%	75.0%	0.0%	75.0%	81.3%	76.0%	80.0%
Nonsense Mutation	0.0%	33.3%	0.0%	0.0%	0.0%	0.0%	4.0%	3.3%
Splice Site	0.0%	0.0%	0.0%	0.0%	0.0%	6.3%	4.0%	0.0%
**SOCS1**								
Frame Shift Deletion	0.0%	0.0%	0.0%	0.0%	0.0%	0.0%	25.0%	50.0%
Frame Shift Insertion	0.0%	0.0%	0.0%	0.0%	0.0%	100.0%	0.0%	0.0%
Missense Mutation	100.0%	0.0%	0.0%	0.0%	100.0%	0.0%	75.0%	50.0%
**STAT3**								
Frame Shift Deletion	0.0%	0.0%	100.0%	0.0%	0.0%	0.0%	7.1%	9.1%
Frame Shift Insertion	0.0%	50.0%	0.0%	0.0%	0.0%	33.3%	7.1%	0.0%
In Frame Deletion	0.0%	0.0%	0.0%	0.0%	11.1%	0.0%	0.0%	0.0%
In Frame Insertion	0.0%	0.0%	0.0%	0.0%	0.0%	0.0%	7.1%	0.0%
Missense Mutation	0.0%	50.0%	0.0%	0.0%	66.7%	33.3%	78.6%	81.8%
Nonsense Mutation	0.0%	0.0%	0.0%	0.0%	11.1%	33.3%	0.0%	0.0%
Splice Site	0.0%	0.0%	0.0%	0.0%	11.1%	0.0%	0.0%	9.1%
**STAT5A**								
Frame Shift Deletion	0.0%	50.0%	0.0%	0.0%	33.3%	0.0%	0.0%	0.0%
Frame Shift Insertion	0.0%	50.0%	0.0%	0.0%	0.0%	0.0%	14.3%	9.1%
In Frame Deletion	0.0%	0.0%	50.0%	0.0%	0.0%	16.7%	0.0%	0.0%
Missense Mutation	0.0%	0.0%	50.0%	100.0%	66.7%	66.7%	85.7%	63.6%
Nonsense Mutation	0.0%	0.0%	0.0%	0.0%	0.0%	16.7%	0.0%	0.0%
Splice Site	0.0%	0.0%	0.0%	0.0%	0.0%	0.0%	0.0%	27.3%
**STAT5B**								
Frame Shift Deletion	0.0%	50.0%	0.0%	0.0%	80.0%	12.5%	12.5%	36.4%
Frame Shift Insertion	0.0%	33.3%	100.0%	0.0%	0.0%	0.0%	0.0%	27.3%
Missense Mutation	0.0%	16.7%	0.0%	0.0%	20.0%	75.0%	87.5%	27.3%
Nonsense Mutation	0.0%	0.0%	0.0%	0.0%	0.0%	12.5%	0.0%	4.5%
Splice Site	0.0%	0.0%	0.0%	0.0%	0.0%	0.0%	0.0%	4.5%

Table S13. Overall survival patterns associated with JAK–STAT pathway alterations across ancestry, age-at-onset, and FOLFOX treatment strata.

Clinical Subgroup	FOLFOX Status	Ancestry	Age Group	Prognostic Effect of JAK–STAT Alterations	Statistical Significance	Key Observations
2.6.1 EOCRC H/L	Yes	H/L	Early-Onset	No significant association	p = 0.68	Curves overlapped; wide CI due to low mutation count
2.6.2 EOCRC H/L	No	H/L	Early-Onset	No significant association	p = 0.25	Visual separation but confidence intervals overlapped

2.6.3 LOCRC H/L	Yes	H/L	Late-Onset	No significant association	p = 0.24	Minimal curve separation; wide CIs in altered group
2.6.4 LOCRC H/L	No	H/L	Late-Onset	No significant association	p = 0.31	Stable altered curve; overlapping CIs
2.6.5 EOCRC NHW	Yes	NHW	Early-Onset	**Improved survival with alterations**	**p = 0.00083**	Strong curve separation; durable survival advantage
2.6.6 EOCRC NHW	No	NHW	Early-Onset	No significant association	p = 0.072	Mild early separation that reconverged over time
2.6.7 LOCRC NHW	Yes	NHW	Late-Onset	No significant association	p = 0.36	Nearly identical survival trajectories
2.6.8 LOCRC NHW	No	NHW	Late-Onset	**Improved survival with alterations**	**p = 0.017**	Persistent curve separation; slower decline in altered group

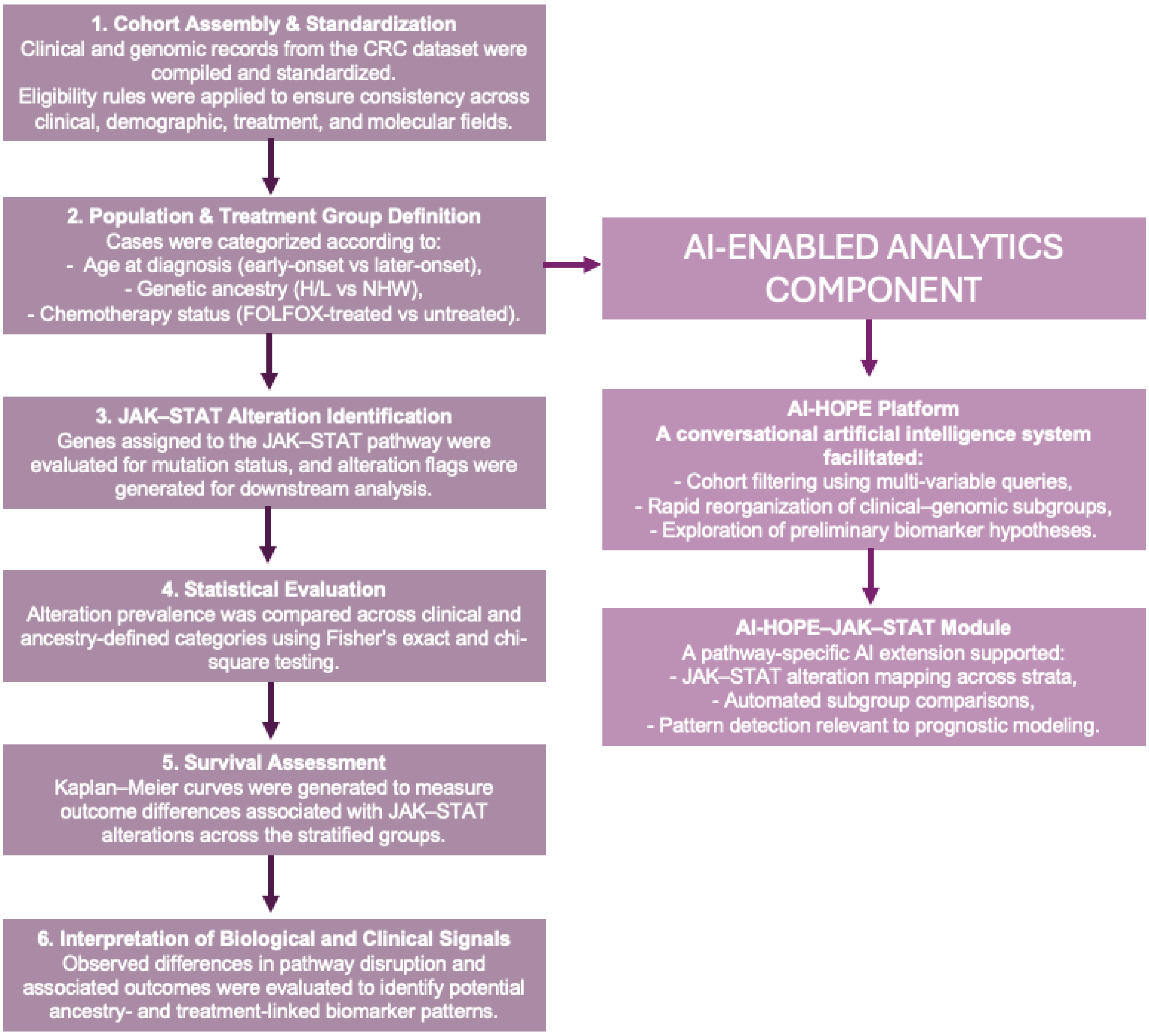

Figure S1. Overview of the study workflow integrating statistical analyses with AI-enabled interrogation of JAK–STAT pathway alterations in colorectal cancer. This diagram summarizes the analytical pipeline used to evaluate JAK–STAT pathway alterations across colorectal cancer cases stratified by age of onset, ancestry, and FOLFOX chemotherapy exposure. The workflow begins with cohort assembly and standardization of clinical and genomic records, followed by definition of population and treatment subgroups. JAK-STAT pathway mutations were identified and flagged for downstream comparison. Conventional analyses, including Fisher's exact and chi-square testing were used to assess alteration prevalence across strata, and Kaplan–Meier survival estimation evaluated prognostic differences. Biological and clinical signals were interpreted to identify ancestry- and treatment-specific biomarker patterns. In parallel, an AI-enabled analytics component supported data exploration and subgroup resolution. The AI-HOPE platform facilitated natural language–driven cohort refinement and hypothesis generation, while the pathway-specific AI-HOPE–JAK–STAT module enabled automated alteration mapping and prognostic pattern detection. Together, these analytic streams provided a combined statistical and artificial intelligence framework to characterize JAK–STAT alterations in early-onset and late-onset colorectal cancer.

(a)

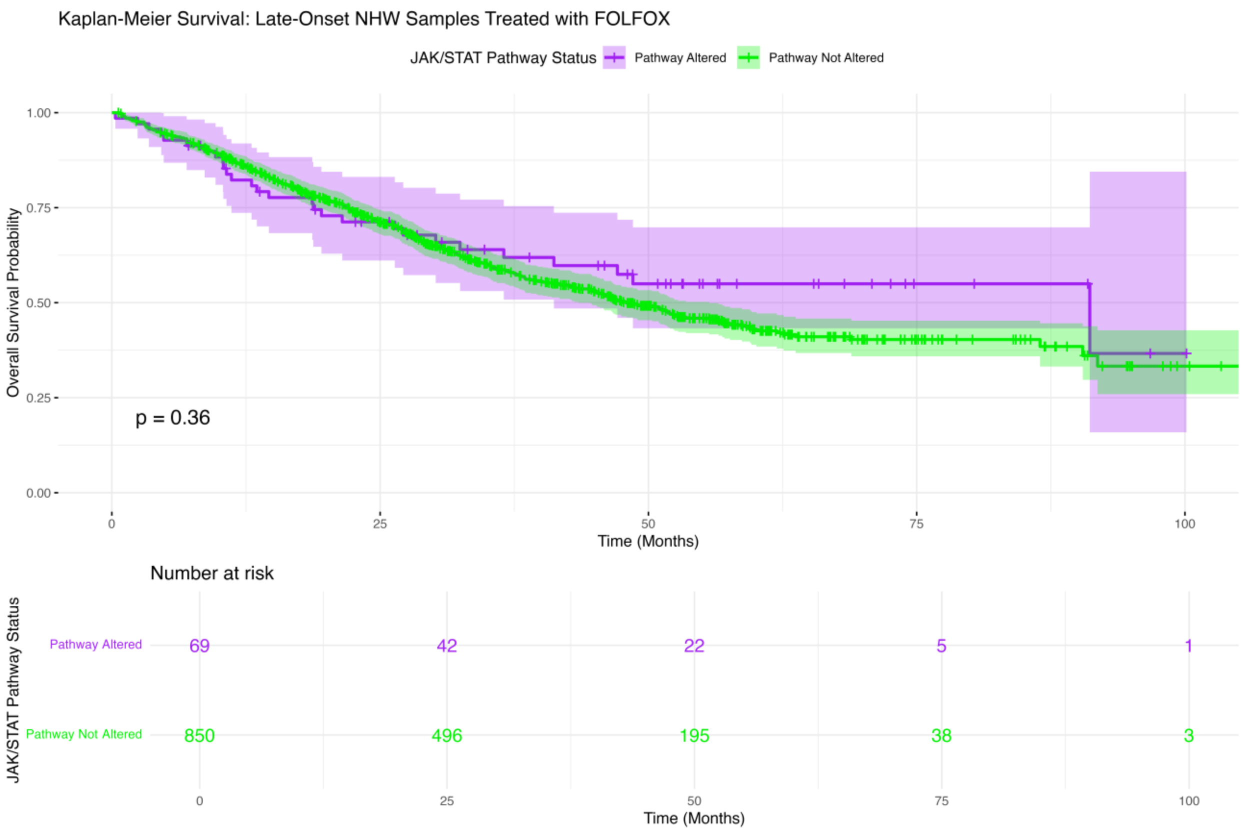

(b)

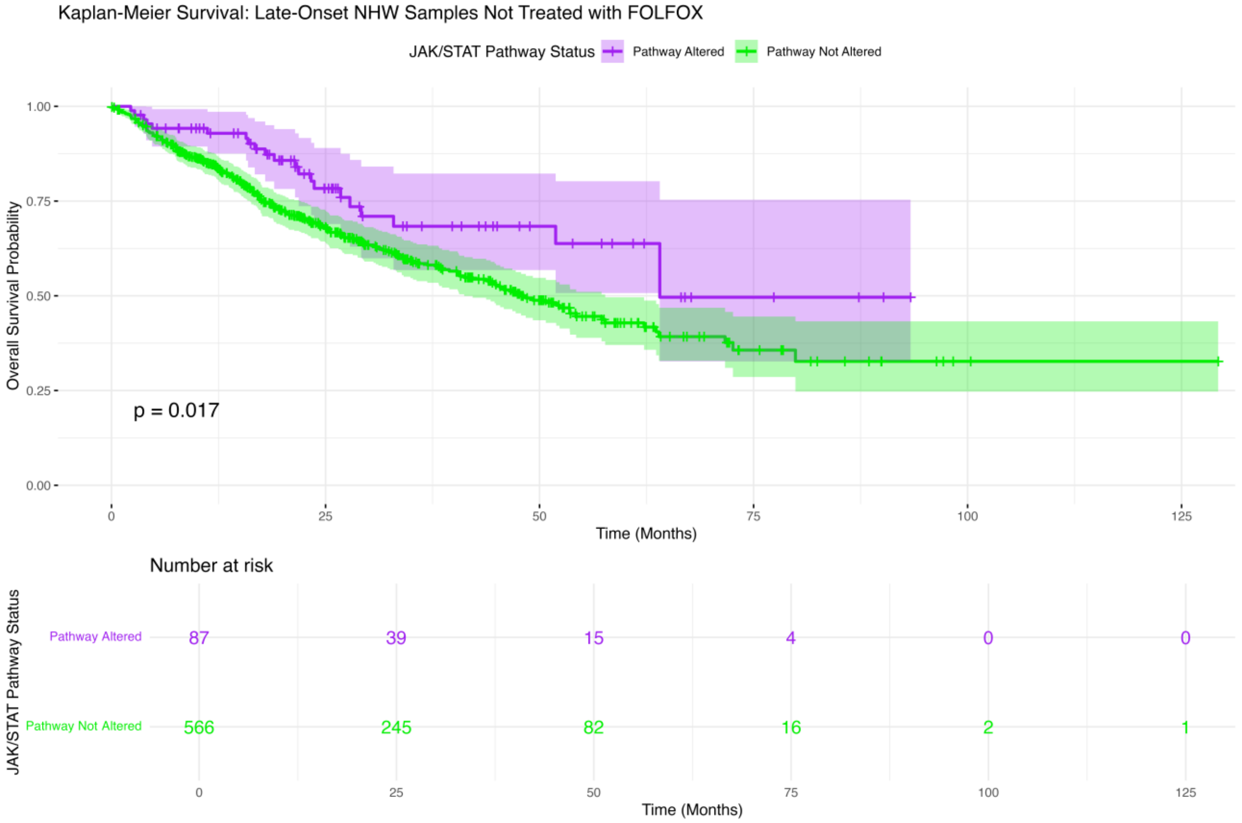

Figure S2. Kaplan-Meier survival curves evaluating the impact of JAK-STAT pathway alterations on overall survival in colorectal cancer (CRC), stratified by age group, ancestry, and FOLFOX treatment. Survival outcomes are presented for two subgroups: (a) late-onset NHW

patients who received FOLFOX therapy, and (b) late-onset NHW patients who did not receive FOLFOX.

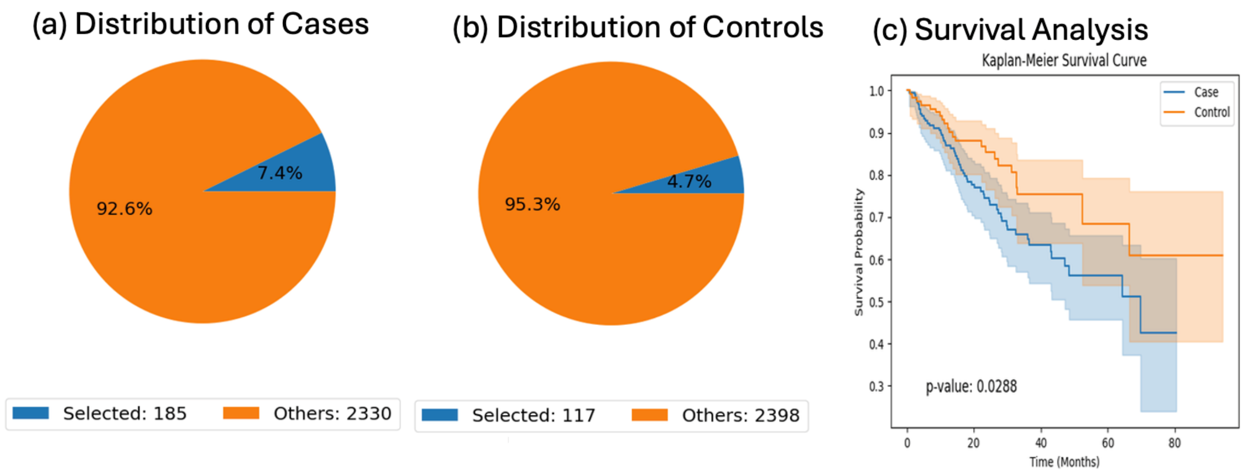

Figure S3. AI-guided cohort construction and survival comparison for early-onset Non-Hispanic White (NHW) colorectal cancer (CRC) patients not treated with FOLFOX, stratified by RTK/RAS pathway alteration status. Using natural language-driven querying, the AI-HOPE-JAK-STAT platform automatically identified case and control cohorts based on user-defined clinical and genomic criteria. (a) The case cohort consisted of early-onset (EO) NHW CRC patients not treated with FOLFOX who harbored RTK/RAS pathway alterations (n = 185), representing 7.4% of all samples within this subgroup. (b) The control cohort included EO NHW patients not treated with FOLFOX and lacking RTK/RAS pathway alterations (n = 117), corresponding to 4.7% of samples in this category. (c) Kaplan-Meier overall survival (OS) analysis comparing altered versus non-altered groups revealed a statistically significant difference (log-rank p = 0.0288), with pathway-altered patients demonstrating reduced survival probabilities over time. Shaded regions indicate 95% confidence intervals. These AI-derived cohort selections and survival trends highlight the potential prognostic relevance of RTK/RAS pathway alterations in EO NHW patients who did not receive FOLFOX.

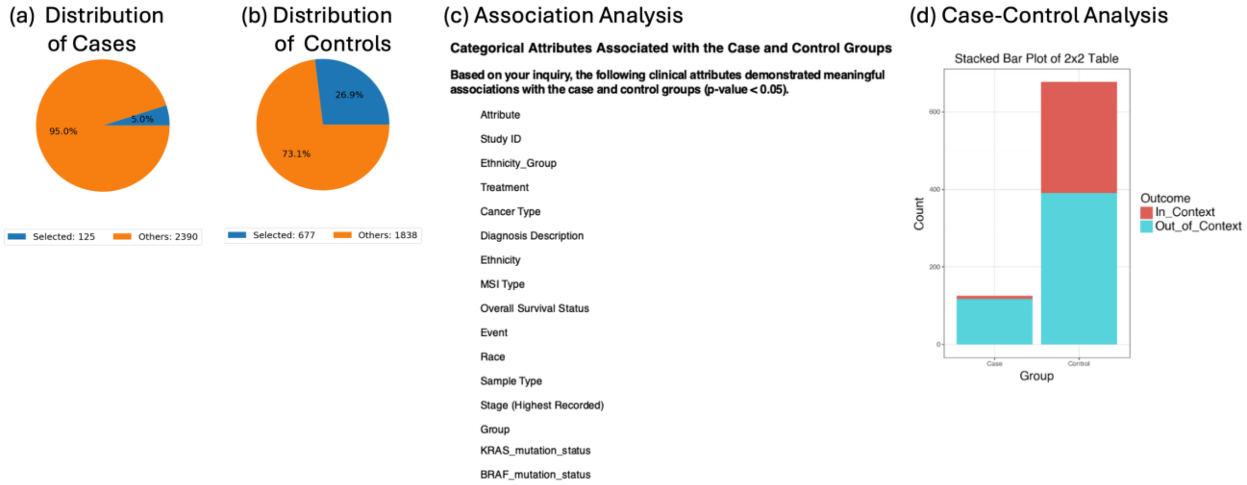

Figure S4. AI-guided identification of clinical attributes distinguishing case and control cohorts in the AI-HOPE-JAK-STAT exploratory analysis. Using the AI-HOPE-JAK-STAT platform, case samples (n = 125) were compared with control samples (n = 677) to identify categorical clinical features meaningfully associated with cohort membership. Panels (a) and (b) display the distribution of selected (in-context) versus unselected samples within the case and control groups, respectively. The case cohort contained 95.0% unselected samples and 5.0% selected samples, whereas the control cohort contained 73.1% unselected and 26.9% selected samples, illustrating a higher enrichment of selected samples within the control group. Panel (c) summarizes the categorical attributes identified by the AI-HOPE-JAK-STAT system as significantly associated with case-control classification ($p < 0.05$). These attributes included Study ID, Ethnicity_Group, Treatment, Cancer Type, Diagnosis Description, MSI Type, Overall Survival Status, Event, Race, Sample Type, Stage (highest recorded), Group, and key mutation variables such as KRAS and BRAF mutation status. These associations provide data-driven hypotheses regarding clinical and molecular variables contributing to differences between the two groups. Panel (d) shows the stacked 2×2 bar plot comparing the distribution of in-context and out-of-context samples across the case and control cohorts. This visualization highlights the disproportionate representation of selected samples within the control group, suggesting potentially meaningful clinical or molecular distinctions requiring further confirmatory statistical evaluation. Together, these AI-guided exploratory findings demonstrate the capacity of AI-HOPE-JAK-STAT to rapidly surface clinically relevant categorical differences between cohorts, supporting downstream hypothesis generation and deeper statistical modeling.

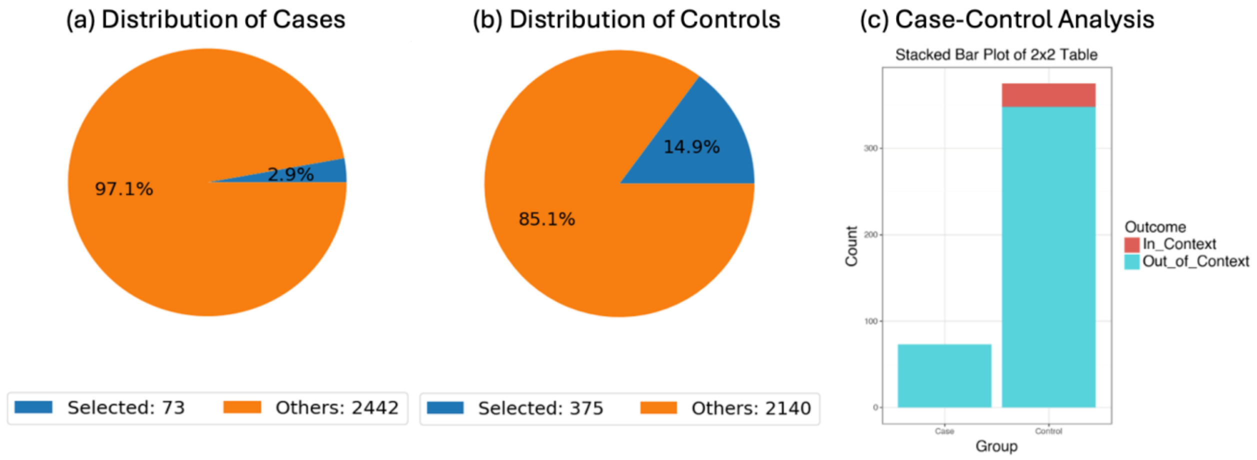

Figure S5. AI-assisted comparison of BRAF mutation prevalence between early-onset Hispanic/Latino (H/L) and Non-Hispanic White (NHW) colorectal cancer (CRC) patients treated with FOLFOX.

Using the AI-HOPE-JAK-STAT platform, early-onset H/L CRC patients (case cohort; n = 73) were compared with early-onset NHW patients (control cohort; n = 375) to evaluate differences in BRAF mutation frequency under identical treatment conditions. The pie charts illustrate the proportion of selected samples (BRAF-mutated; blue) versus unselected samples (non-mutated; orange) in each cohort, revealing a substantially lower fraction of BRAF-mutant cases in the H/L group. The stacked bar plot on the right visualizes “In_Context” (BRAF-mutated) and “Out_of_Context” samples across case and control cohorts. Fisher’s exact test demonstrated a statistically significant difference between groups (p = 0.036), with an odds ratio of 0.0 (95% CI: 0.005-1.464). Only 0.68% of H/L cases were BRAF-mutated compared with 7.2% of NHW controls, indicating that early-onset NHW patients treated with FOLFOX were more likely to harbor BRAF mutations than their H/L counterparts. These findings highlight ancestry-associated molecular differences in early-onset CRC and underscore the utility of AI-driven interrogation for uncovering clinically relevant genomic disparities.

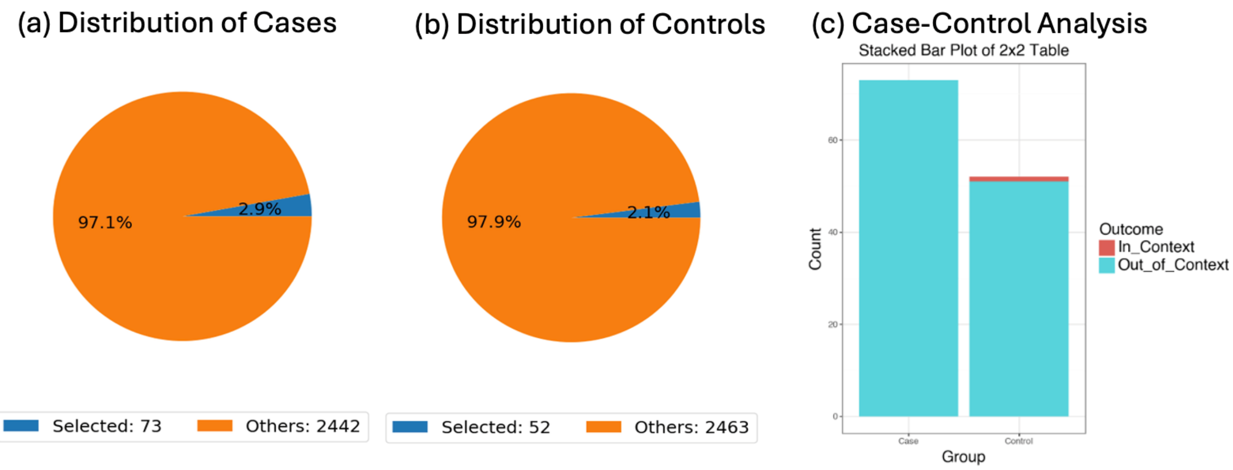

Figure S6. AI-assisted comparison of ERBB2 mutation frequency between early-onset Hispanic/Latino (H/L) colorectal cancer (CRC) patients treated with FOLFOX and early-onset H/L patients not treated with FOLFOX.
The AI-HOPE-JAK-STAT platform was used to define case and control cohorts based on user-specified clinical and treatment parameters. The case cohort consisted of early-onset H/L CRC patients who received FOLFOX (n = 73), while the control cohort included early-onset H/L patients who did not receive FOLFOX (n = 52). ERBB2 mutation status was used as the in-context criterion for comparison. The pie charts illustrate the proportion of selected (in-context) versus unselected (out-of-context) samples within each cohort. The stacked bar plot summarizes the number of ERBB2-mutated and non-mutated samples across case and control groups. Fisher's exact test showed no statistically significant difference in ERBB2 mutation prevalence between treated and untreated early-onset H/L patients (p = 0.864), with an odds ratio of 0.0 (95% CI: 0.012-10.61). These results indicate that ERBB2 mutation frequency does not differ meaningfully by FOLFOX treatment status within early-onset H/L CRC patients.
